# Assessment of false negative rates of lactate dehydrogenase-based malaria rapid diagnostic tests for *Plasmodium ovale* detection

**DOI:** 10.1371/journal.pntd.0007254

**Published:** 2019-03-11

**Authors:** Jianxia Tang, Feng Tang, Hongru Zhu, Feng Lu, Sui Xu, Yuanyuan Cao, Yaping Gu, Xiaoqin He, Huayun Zhou, Guoding Zhu, Jun Cao

**Affiliations:** 1 NHC Key Laboratory of Parasitic Disease Control and Prevention, Jiangsu Provincial Key Laboratory on Parasite and Vector Control Technology (Jiangsu Institute of Parasitic Diseases), Wuxi, China; 2 Center for Global Health, School of Public Health, Nanjing Medical University, Nanjing, China; 3 Public Health Research Center, Jiangnan University, Wuxi, China; Mahidol University, THAILAND

## Abstract

Currently, malaria rapid diagnostic tests (RDTs) are widely used for malaria diagnosis, but test performance and the factors that lead to failure of *Plasmodium ovale* detection are not well understood. In this study, three pLDH-based RDTs were evaluated using cases in China that originated in Africa. The sensitivity of Wondfo Pf/Pan, CareStart pLDH PAN and SD BIOLINE Pf/Pan in *P*. *ovale* detection was 70, 55 and 18%, respectively. CareStart was worse at detecting *P*. *o*. *curtisi* (36.5%) than at detecting *P*. *o*. *wallikeri* (75.0%), and SD could not detect *P*. *o*. *curtisi*. The overall detection ratio of all three RDTs decreased with parasite density and pLDH concentration. Wondfo, CareStart and SD detected only 75.0, 78.1 and 46.9% of the *P*. *ovale* cases, respectively, even when the parasitemia were higher than 5000 parasites/μL. Subspecies of *P*. *ovale* should be considered while to improve RDT quality for *P*. *ovale* diagnosis to achieve the goal of malaria elimination.

## Author summary

*Plasmodium ovale (P*. *ovale)* are under-estimated and overshadowed by other malaria parasites in tropical countries, which can cause chronic infections that last from months to years. The chronic infection caused by *P*. *ovale* should be of concern in the context of the long-term goal of eliminating malaria. Rapid diagnostic tests (RDTs) is one of the WHO recommended tools to confirm the infection of *plasmodium* parasites, which can distinguish *Plasmodium falciparum* and non-*falciparum* species as well. However, little is known about their performance detecting *P*. *ovale*, and the factors that affect the efficiency of RDTs in the detection of *P*. *ovale* have not been systemically investigated. This study suggested that the performance of the three pLDH-based RDTs for *P*. *ovale* detection was not optimal, the low parasite density and pLDH concentration contributed to the failure of the RDT test for *P*. *ovale*. It provided information for the application of malaria RDTs in the field and for research and development to improve RDTs for malaria diagnosis.

## Introduction

*Plasmodium ovale* has a wide geographic distribution across tropical countries, especially in Africa, Asia and some Western Pacific islands [1]. *P*. *ovale* has been overshadowed by other human malaria parasites in the field of medicine and medical research because of the relatively low morbidity and infections can be easily treated with conventional antimalarial drugs [2]. Although considered mild, *P*. *ovale* can cause acute respiratory distress syndrome and acute renal failure [3]. In addition, in the context of the long-term goal of eliminating malaria, it is becoming important to diagnose *P*. *ovale* in a timely manner. *P*. *ovale*, which shares with *P*. *vivax* the ability to form hypnozoites [4, 5], can cause chronic infections that may last from months to even years.

Rapid and reliable diagnosis is one of the key factors for malaria control and elimination. Accurate identification of *Plasmodium* infections is critical for administration of a targeted therapy, having a positive impact on patient health, disease management, and preventing transmission risk. Accurate diagnosis of malaria is needed to prevent the emergence and spread of drug resistant strains and to reduce the cost of medicine. The gold standard for malaria diagnosis remains the examination of Giemsa-stained smears by microscopy. This technique requires considerable training and experience. It is very challenging to maintain the capacity for microscopic examination in areas where malaria is being or has been eliminated [6]. In addition, *P*. *ovale* infection is difficult to diagnose microscopically owing to the generally low parasite density in patients and the morphology of *P*. *ovale* resembles that of *P*. *vivax*.

Malaria rapid diagnostic tests (RDTs) allow many countries to provide access to accurate malaria diagnosis even in the most remote areas by means of a simple-to-use, point-of-care test [7]. RDTs are easy to use and require no specific training or equipment. The results are visually readable as colored lines on a strip, and no special expertise is required. In recent decades, RDTs have replaced microscopy as the method of choice for diagnosing malaria in various settings. Reported sensitivities vary among different RDTs but are generally good for the detection of *Plasmodium falciparum* [8–11]. However, the sensitivity of RDTs for *P*. *ovale* detection is much lower, only 5.5% to 80%, with a sharp decrease observed at parasite densities lower than 500 parasites /μL [8–9, 11–13]. Based on a few assessments with a very limited number (n = 69–76) of tested samples, failure of *P*. *ovale* detection by RDTs has been reported [12, 14–15]. Malaria RDTs exhibited suboptimal performance in the detection of *P*. *ovale* infections [16], but the factors that affect the efficiency of RDTs in the detection of *P*. *ovale* have not been systemically investigated.

Targets of malaria RDTs are specific antigens of one or more *Plasmodium* species, such as histidine-rich protein (HRP2), lactate dehydrogenase (LDH), and aldolase. Among these antigens, *Plasmodium*-specific lactate dehydrogenase (pan-pLDH) is commonly used as a target of RDTs to detect all human *Plasmodium* species [14]. In addition, *P*. *ovale* is comprised of two genetic subspecies, namely, *P*. *ovale curtisi* and *P*. *ovale wallikeri*, and genetic variations based on *P*. *ovale* LDH gene polymorphism could also be involved in RDT failure [17]. We hypothesized that parasite density, level of pLDH, subspecies of *P*. *ovale* or polymorphism of pLDH gene might be involved in the failure of *P*. *ovale* detection by pLDH-based RDTs.

In this study, the performance of three pLDH-based RDTs (Wondfo diagnostic kit for malaria (Pf/pan) (colloidal gold), CareStart Malaria pLDH (PAN), and SD BIOLINE Malaria Ag Pf/Pan) for *P*. *ovale* detection were retrospectively evaluated with blood samples from returned international travelers and laborers from Africa to China. Moreover, the possible factors affecting RDT detection, i.e., parasite density, pLDH concentration, genetic subspecies and pLDH gene polymorphism, were investigated.

## Methods

### Ethics approval and consent to participate

This study was reviewed and approved by the Institutional Ethics Committee of Jiangsu Institute of Parasitic Diseases (JIPD) (IRB00004221). All participants were adults and written informed consent was obtained from all participants before the interview or evaluation.

### Patients and samples

Blood samples were selected from venous EDTA-blood samples stored at -70°C, which were obtained from febrile patients at the clinics of local hospitals in Jiangsu province, China, and sent to the provincial reference laboratory. The patients were international travelers and laborers from African countries. The diagnosis of *P*. *ovale* was determined by both microscopy and confirmed by a nested PCR assay using the following commonly used protocols [18].

### Microscopic examination

Thick and thin blood films were prepared from peripheral blood. Blood smears were stained with 3% Giemsa for 30 min at room temperature to identify parasites. Smears were analyzed by experienced microscopists at the JIPD. The standard method recommended by the World Health Organization (WHO) was used to estimate the number of circulating parasites per μL of blood. Parasite density was determined by counting the parasites and leucocytes, assuming 8,000 leucocytes /μL [19]. All the slides were subjected to double-blind verification by another independent microscopist, and the results were combined.

### Rapid diagnostic tests for *P*. *ovale* detection

Three pLDH-based RDTs were used in this study: the Wondfo diagnostic kit for malaria (Pf/pan) (colloidal gold) (Guangzhou Wondfo Biotech Co., Ltd.; lot W05440903WC), CareStart Malaria pLDH (PAN) (Access Bio, Inc.; lot MN13G01) and SD BIOLINE Malaria Ag Pf/Pan (Standard Diagnostics Inc.; lots 05ED14111 and 05ED15003). The RDTs were selected based on the WHO/FIND malaria RDT performance evaluations and national guidelines of China. All of the RDTs were packed and sealed individually with desiccant and used immediately after opening and were performed based on the instructions of the manufacturers. The major target antigens of these three RDTs were pLDH, which are specific for all human-associated *Plasmodium* species. Five microliters of stored whole-blood samples were added to the pad, and three to four drops of specific lysis agent were added. The RDT result was read in 15–20 min according to the manufacturer’s instructions and immediately recorded by one person, a second person read the results 5 min later after the first person and was blinded to the initial reading. In the event of discordant results, a third person read the test blindly also and final results was the most common reading. The test was considered valid when the control line on the immunochromatographic test strip was visible. For the Pf/Pan test device (Wondfo diagnostic kit for malaria (Pf/pan) and SD BIOLINE Malaria Ag Pf/Pan), the result was recorded as non-*falciparum* only when the pan-pLDH line was positive. For the pLDH PAN test device (CareStart Malaria pLDH (PAN)), the presence of two colored bands (one band in the control and another band in the test) indicated a positive result for *Plasmodium* infection. All three RDTs were performed once.

### Assessment of pLDH optical density levels in *P*. *ovale* samples

Quantitative levels of pLDH antigens in *P*. *ovale* samples were determined using the Quantimal pLDH Malaria CELISA test (Cellabs), a sandwich ELISA for the detection of human *Plasmodium* pLDH. All *P*. *ovale* blood samples were tested in triplicate, and the manufacturer’s instructions were followed. Plates were read on a Zenyth 340 microplate spectrophotometer (Autobio) at 450 nm, with a reference wavelength of 620 nm. The reading was completed within 30 min after the stop solution was added. The mean optical density (OD) was calculated with the cut-off value as the means plus three standard deviations (SDs) of the wells containing healthy human blood alone.

### DNA extraction and *P*. *ovale* subspecies detection

Genomic DNA was extracted from 200 μL of whole-blood samples of *P*. *ovale-*infected patients using a QIAamp Blood Mini Kit (Qiagen) according to the manufacturer’s instructions. DNA extracted from healthy individuals living in nonendemic areas was used as a negative control in the amplification process. The real-time TaqMan PCR (qPCR) assay was used to detect *P*. *ovale* subspecies as described in a previous publication [20]. Amplification was performed with the following set of primers: POF (5′-ATAAACTATGCCGACTAGGTT-3′) and POR (5′-ACTTTGATTTCTCATAAGGTACT-3′). The probe pPOW HEX-AATTCCTTTTGGAAATTTCTTAGATTG-BHQ1 was used for detection of *P*. *o*. *wallikeri*, and pPOC FAM-TTCCTTTCGGGGAAATTTCTTAGA-BHQ1 was used for detection of *P*. *o*. *curtisi*. The qPCRs were carried out using LightCycler TaqMan Master (Roche, Germany) on a Roche LightCycler 480 (Roche, Germany) under the following conditions: one step at 95°C for 10 min; 45 cycles at 95°C for 15 sec and 60°C for 60 sec; and a final step at 4°C for 10 sec.

### pLDH gene PCR amplification and DNA sequencing

Nucleotide sequences corresponding to *P*. *ovale* LDH genes were amplified with the primers LDHovD21 (5′-GTTCTCGTTGGTCAGGAATGATA-3′) and LDHovC915 (5′-GGCATCATCAAACATCTTCTTTTCT-3′) by conventional PCR using Dream Taq Green PCR Master Mix (Thermo Scientific). Primer design and PCR conditions were based on a previous publication [20]. The PCR products were sequenced by Genescript Biological Technology Co., Ltd. (Nanjing, China). Nucleotide sequences of *P*. *ovale* LDHs were aligned using BioEdit software and compared with available *P*. *ovale* sequences in GenBank (accession number AY486058) and a paper by Bauffe et. al.[20]. Moreover, amino acid sequences were derived using GeneDoc software and also compared with available *P*. *ovale* sequences.

### Statistical analysis

The RDT results for *P*. *ovale* detection were compared with each other, and sensitivity was calculated with 95% confidence intervals (CIs) by STATA (version 12.0). Categorical variables were determined by Chi-squared tests, with Fisher’s exact correction applied when the expected frequency in any cell was 5 or less. Correlation analyses between parasite density and pLDH OD level were performed as Pearson’s correlation analyses. A Pearson *r* value greater than 0.6 was considered a strong correlation. *P* values less than 0.05 were considered significant for all statistical analyses.

## Results

### Sample collection

A total of 100 samples containing *P*. *ovale* parasites were studied. The samples had been collected from Feb 2012 to June 2015. The average age of the patients was 41.27 years (range, 22–58 years), and all the *P*. *ovale* infections were acquired in Africa. A majority (38/100) of the *P*. *ovale* samples were from Equatorial Guinea, followed by Nigeria (18/100), Angola (17/100) and the Republic of Congo (9/100) (Table 1).

**Table 1:** Origins the of *P*. *ovale* infection acquired in Africa.

Countries	No. of samples	No. of *P ovale curtisi*	No. of *P*. *o*. *wallikeri* s
Equatorial Guinea	38	24	14
Nigeria	18	10	8
Angola	17	5	12
Congo	9	5	4
Congo, DRC	3	2	1
Guinea	1	0	1
Gabon	5	2	3
Cameroon	2	2	0
Liberia	1	0	1
Mozambique	2	1	1
South Sudan	1	0	1
Sierra Leone	1	0	1
Uganda	1	0	1
Chad	1	1	0
**Total**	**100**	**52**	**48**

### False negative rates of pLDH-based RDTs for *P*. *ovale* detection

The performances of the three pLDH-based RDTs (Wondfo Pf/pan, CareStart pLDH (PAN) and SD Pf/Pan) were compared for *P*. *ovale* detection. Of all the 100 confirmed *P*. *ovale* samples, 70 tested positive with the Wondfo Pf/Pan device, 55 tested positive with the CareStart pLDH device, and only 18 tested positive with the SD Pf/Pan device (Table 2). All three RDTs exhibited very high false negative rates for *P*. *ovale* detection.

**Table 2:** Results for the detection of *P*. *ovale* infection by the three different RDTs.

RDT	No. of samples	No. of positive samples	No. of negative samples	Sensitivity (%)
**Wondfo Pf/pan**	100	70	30	70 (60.0–78.8)
**CareStart pLDH (PAN)**	100	55	45	55 (44.7–65.0)
**SD Pf/Pan**	100	18	82	18 (11.0–26.9)

Sensitivity is presented as a percentage (95% confidence interval; CI)

### Some RDTs failed to detect *P*. *ovale curtisi*

To investigate whether the subspecies of *P*. *ovale* and the variations in *P*. *ovale* LDH polymorphism were associated with the sensitivity of the three RDTs for *P*. *ovale* detection, a real-time TaqMan PCR (qPCR) assay was carried out for subspecies determination, and the pLDH gene was sequenced for polymorphism analysis. Among the 100 *P*. *ovale* samples, 52 were *P*. *o*. *curtisi* and 48 were *P*. *o*. *wallikeri*, as determined by qPCR. The two subspecies exhibited no difference in parasitemia and pLDH levels (*P*>0.05).

There was no significant difference in the sensitivity of the detection of the two subspecies by the Wondfo Pf/Pan device (*χ*^2^ = 0.49, *P* = 0.485). A higher sensitivity was observed for *P*. *o*. *wallikeri* than that for *P*. *o*. *curtisi* (approximately 75% vs 36.5%) with the CareStart Pan device with statistically significance (*χ*^2^ = 14.92, *P*<0.0001). On the other hand, the SD Pf/Pan device could not detect *P*. *o*. *curtisi* at all, and a high false negative rate (62.5%) was observed for *P*. *o*. *wallikeri* detection with this device (*χ*^2^ = 20.88, *P*<0.0001, Fisher’s exact<0.0001 and one-sided Fisher’s exact<0.0001) (Table 3).

**Table 3:** Comparison of the sensitivity of the three RDTs in the detection of *P*. *ovale curtisi* and *P*. *ovale wallikeri*.

*P*. *ovale*	No. of samples	Wondfo			CareStart			SD		
		No. of positive samples	No. of negative samples	Sensitivity (%)	No. of positive samples	No. of negative samples	Sensitivity (%)	No. of positive samples	No. of negative samples	Sensitivity (%)
***P*. *o*. *curtisi***	52	38	14	73.1(59.0–84.4)	19	33	36.5(23.6–51.0)	0	52	0(0–6.8)*
***P*. *o*. *wallikeri***	48	32	16	66.7(51.6–79.6)	36	12	75.0(60.4–86.4)	18	30	37.5(24.0–52.6)

Sensitivity is presented as a percentage (95% confidence interval; CI);

*one-sided, 97.5% CI

The amplified *P*. *ovale* LDH gene yielded approximately 890 base pairs, coding for 294 amino acids. A total of 100 of the amplified genes were sequenced to analyze the genetic variations in the *P*. *ovale* LDH gene using Clustal W2 software. No nucleotide substitution was detected within the sequences of each subspecies compared to the reference sequences (*P*. *o*. *curtisi* from GenBank (AY486058) and *P*. *o*. *wallikeri* from a paper by Bauffe et. al.). There were twenty-four single nucleotide polymorphisms (SNPs) between the two subspecies, and three of these SNPs were nonsynonymous mutations (S143P, N168K, I204V), which was consistent with the results of a previous study [20].

### The sensitivity of the RDTs for *P*. *ovale* detection was associated with parasite densities

The performance of each RDT was evaluated according to parasite density levels. Based on *P*. *ovale* parasite densities, the 100 samples were divided into three groups. For the Wondfo Pf/Pan device, the sensitivity for cases with parasite densities greater than 500 parasites/μL (75.4% and 75%) was higher than that for cases with densities lower than 500 parasites/μL (27.3%), and this difference was statistically significant (*χ*^2^ = 10.75, *P*<0.05, Fisher’s exact = 0.007). However, for the SD Pf/Pan device, regardless of parasite density, the sensitivity was less than 50%, and the cases with parasite densities lower than 500 parasites/μL could not be detected with this device and a significant difference was observed (*χ*^2^ = 26.76, *P*<0.0001, Fisher’s exact<0.0001). The sensitivity of the CareStart Pan device reached 78.1% for only those cases that had parasite densities greater than 5000 parasites/μL, and a significant difference was observed (*χ*^2^ = 10.49, *P* = 0.005, Fisher’s exact = 0.005) (Table 4).

**Table 4:** Comparison of the sensitivity of the three RDTs in the detection of *P*. *ovale* categorized by parasite density.

**Parasite density (parasites/μL)**	**No. of samples**	**Wondfo**			**CareStart**			**SD**		
		No. of positive samples	No. of negative samples	Sensitivity (%)	No. of positive samples	No. of negative samples	Sensitivity (%)	No. of positive samples	No. of negative samples	Sensitivity (%)
**≤ 500**	11	3	8	27.3(6.0–60.9)	4	7	36.4(10.9–69.2)	0	11	0(0–28.5)*
**501–5000**	57	43	14	75.4(62.2–85.9)	26	31	45.6(32.4–59.3)	3	54	5.3(10.9–14.6)
**≥ 5001**	32	24	8	75.0(56.6–88.5)	25	7	78.1(60.0–90.7)	15	17	46.9(29.1–65.3)

Sensitivity is presented as a percentage (95% confidence interval; CI);

*one-sided, 97.5% C

### The sensitivity of pLDH-based RDTs for *P*. *ovale* detection was correlated with pLDH concentration

To determine the relationship between pLDH concentration and the sensitivity of the three RDTs for *P*. *ovale* detection, the *P*. *ovale* samples were divided into three groups according to the pLDH OD levels, and the sensitivity of the three RDTs was evaluated in each group. For the Wondfo Pf/Pan and CareStart Pan device, the sensitivity for *P*. *ovale* detection reached 100% and 89.7% when the pLDH OD levels in the samples were more than 0.5; the sensitivity increased with the pLDH levels; and a significant difference was observed (*χ*^2^ = 82.05, *P*<0.0001, Fisher’s exact<0.0001 for Wondfo; *χ*^2^ = 32.09, *P*<0.0001, Fisher’s exact<0.0001 for CareStart). On the other hand, the sensitivity of the SD Pf/Pan device increased with the pLDH levels, and a significant difference was also observed (*χ*^2^ = 38.52, *P*<0.0001, Fisher’s exact<0.0001), but the sensitivity reached only 55.2%, even when the pLDH level was high (>0.5 OD) (Table 5).

**Table 5:** Comparison of the sensitivity of the three RDTs in the detection of *P*. *ovale* categorized by pLDH concentration.

pLDH concentration (OD)	No. of samples	Wondfo			CareStart			SD		
		No. of positive samples	No. of negative samples	Sensitivity (%)	No. of positive samples	No. of negative samples	Sensitivity (%)	No. of positive samples	No. of negative samples	Sensitivity (%)
<0.100	30	2	28	6.7(8.2–22.1)	5	25	16.7(56.4–34.7)	0	30	0(0–11.6)*
0.100–0.500	41	39	2	95.1(83.5–99.4)	24	17	58.5(42.1–73.7)	2	39	4.9(26.3–57.9)
>0.501	29	29	0	100(88.1–100) *	26	3	89.7(72.6–97.8)	16	13	55.2(35.7–73.6)

Sensitivity is presented as a percentage (95% confidence interval; CI);

*one-sided, 97.5% CI

### The pLDH concentration was correlated with parasitemia in *P*. *ovale* samples

**Fig 1:**
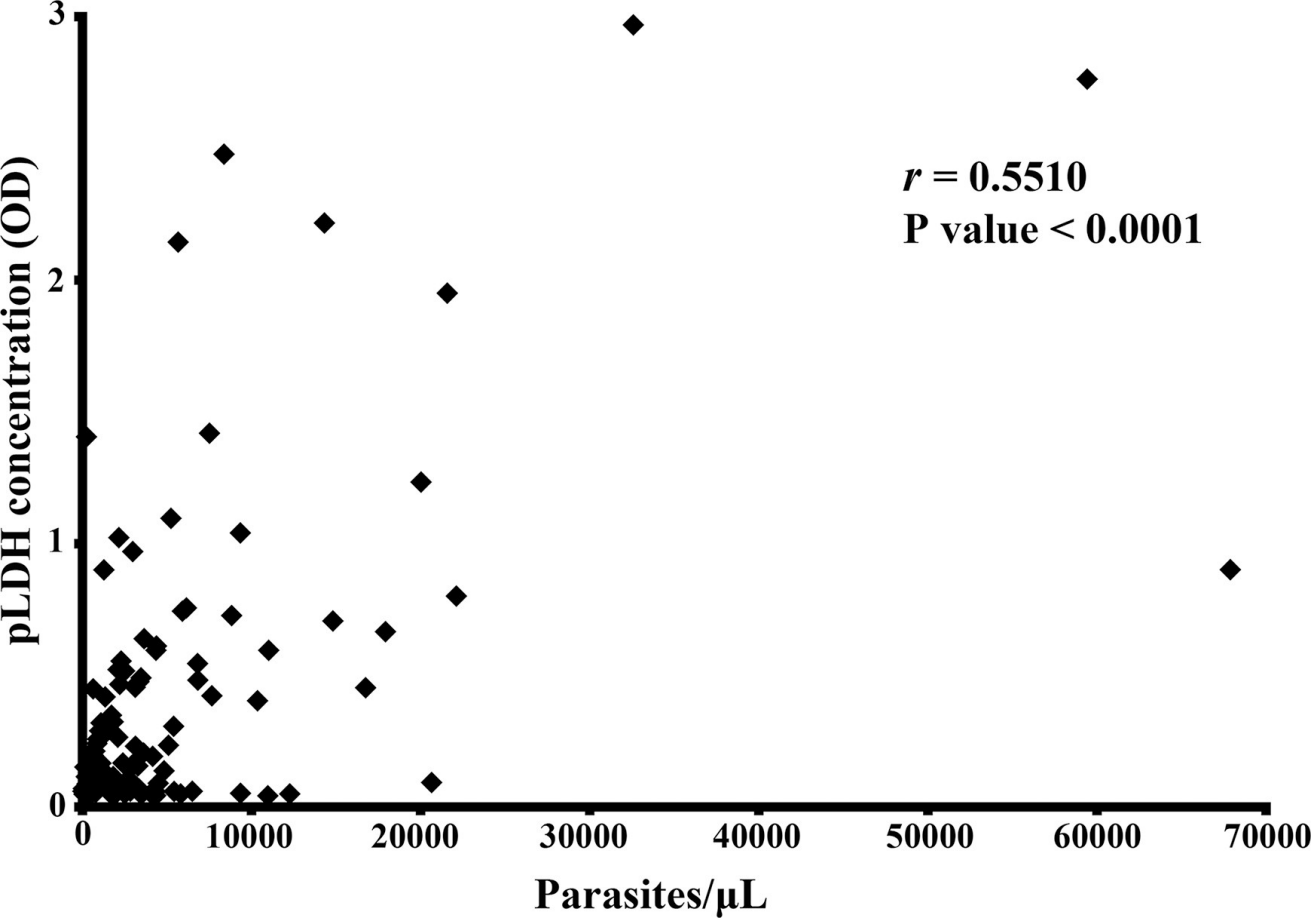
Correlations between parasite density and pLDH levels among Plasmodium ovale isolates (r = 0.5510).

To determine whether the pLDH levels were associated with parasitemia in these *P*. *ovale* samples, the correlation between the pLDH levels and peripheral blood parasitemia was assessed. A moderate correlation was observed between pLDH levels and parasitemia (*r* = 0.5510, *P* value<0.0001). Some disagreement was observed between parasitemia and pLDH levels. Ten cases of *P*. *ovale* samples with parasitemia greater than 10,000 parasites/μL presented low levels of pLDH (OD<1), and five cases with low parasitemia (<10,000 parasites/μL) presented high pLDH levels (OD>1) (Fig 1).

## Discussion

Currently, hundreds of RDTs are available in the global market for malaria diagnosis, and these tests play a very important role in current malaria control/elimination programs. However, the quality of malaria RDTs varies among different companies, even among different lots from the same company. The FIND-WHO global RDT evaluation program was carried out on *P*. *falciparum* and *P*. *vivax* clinical samples (https://www.finddx.org/malaria/) [21], but there is limited information regarding the effectiveness of RDTs in *P*. *ovale* and *Plasmodium malariae* detection. This study evaluated three pLDH-based RDTs (Wondfo Pf/Pan, CareStart PAN, and SD Pf/Pan) for *P*. *ovale* malaria diagnosis using cases of malaria infections acquired in Africa and brought to China. The results showed that the three RDTs performed poorly in the detection of *P*. *ovale*. The most sensitive test, namely, Wondfo Pf/Pan, detected only 70% of the confirmed *P*. *ovale* samples; similar results were obtained in a previous study [9, 11]. In this study, the sensitivity of the SD Pf/Pan test for *P*. *ovale* was only 18%, which was much lower than that observed in previous reports (76.3% to 90.5% and 76.9%) [9, 11]. This discrepancy may be due to differences in product design or product load. The high rate of false negative results of the RDTs in *P*. *ovale* detection is a major challenge for malaria elimination in areas with a prevalence of both *P*. *falciparum* and non-*falciparum* strains.

Normally, the performances of malaria RDTs are analyzed by comparing the values with the parasite density levels observed by microscopic examination. The poor performance of RDTs in the detection of *P*. *ovale* may be explained by low parasite density, but several exceptional cases could not be explained by parasite density alone. All three RDTs had similar limits of detection, and all three exhibited poor performances for infections with parasite densities less than 500 parasites/μL. The sensitivity for the high parasitemia group (higher than 5000 parasites/μL) was also unsatisfactory, which was observed in other studies [8, 9, 11–13].

The pLDH antigen is one of the most promising antigens explored so far and is assumed to be a specific marker for the presence of *Plasmodium* in blood. pLDH does not persist in the blood [22, 23], and the amount of pLDH indicates the metabolic presence of *Plasmodium* parasites due to the low stability of pLDH in the body [24]. In this study, lower sensitivities of all three RDTs were observed in groups with lower pLDH antigen levels. The sensitivity reached 100% and 89.7% when the pLDH OD was greater than 0.5 for the Wondfo Pf/Pan and CareStart Pan test. Thus, the pLDH levels of *P*. *ovale* were one of contributing factors to the variations in the performance of pLDH-based RDTs, which was consistent with the results of a similar study on the detection of *P*. *vivax* with RDTs [24]. On the other hand, the relationship between pLDH levels and parasite density was assessed in this study, and a positive correlation was observed (*r* = 0.5510, *P* value<0.0001). A similar result was also observed for *P*. *vivax* that pLDH levels showed moderate correlation with parasite density (r = 0.4, *P* < 0.05) [24]. Some discrepancies were observed between parasite density and pLDH levels, while similar results were also observed in study of *P*. *vivax* [24] and in a rodent malaria model [25]. There are 36.4% (4/11) cases with low parasitaemia (<500 p/μL) and relatively high pLDH (OD>0.1), while 33.3% (5/15) cases have high parasitaemia (>10,000 p/μL) and relatively low pLDH (OD<0.5). A total of 9 cases (9% of the total population in the study) were not coordinate with the correlation between parasitaemia and pLDH level. Since the evaluation of RDTs is currently based on patients’ parasitaemia levels, while little is known about the targeted biomarkers in clinical patients, which maybe one of the factors that cause the discordant results in the present study.

Except for low parasite density and pLDH levels, the failure of the RDT test for *P*. *ovale* detection is also currently hypothesized to be contributed to the natural variability among tested species. The false negative rate observed for *P*. *o*. *curtisi* was higher than that for *P*. *o*. *wallikeri* (approximately 60% vs 43%, respectively) [20]. No substantial difference was observed with the Wondfo Pf/Pan test between both *P*. *ovale* species detected in this study. With the CareStart Pan device, the false negative rate observed for *P*. *o*. *curtisi* was higher than that for *P*. *o*. *wallikeri* (approximately 75% vs. 36.5%, respectively), which was consistent with the results of a previous study [20]. The SD Pf/Pan device could not detect *P*. *o*. *curtisi* at all, while a high false negative rate (62.5%) for *P*. *o*. *wallikeri* was observed. As genetic variations based on *P*. *ovale* LDH could be involved in RDT failure [17], *P*. *ovale* LDH gene polymorphism was evaluated for *P*. *o*. *curtisi* and *P*. *o*. *wallikeri*, and no nucleotide substitution was observed in either subspecies. Similar result was observed on *P*. *falciparum ldh* sequences, which also highly conserved with haplotype and nucleotide diversity value 0.203 and 0.0004 [26]. However, the isoforms of pLDH maybe different and the binding site of the antibody is unknown, these factors could be in involved in the failure of the *P*. *ovale* detection by RDTs.

The present study has its limitation. For the retrospective samples, it was impossible to explore the cause of discordant results, such as the sensitivity for the high parasitemia group (higher than 5000 parasites/μL) was unsatisfactory. Another limitation was that readers of RDTs were not blinded to the results of microscopy and PCR, which will cause the subjective bias. Furthermore, as frozen blood samples were applied in the study and an influence of sample storage, such as cell lysis and decreased level of pLDH caused by frozen-thraw, cannot be excluded.

### Conclusion

The results of this study suggested that the performance of the three pLDH-based RDTs for *P*. *ovale* detection was not optimal. The low parasite density and pLDH concentration contributed to the failure of the RDT test for *P*. *ovale*. The subspecies of *P*. *ovale* can affect the sensitivity of the detection of *P*. *ovale* for the CareStart Pf/Pan and SD PAN RDTs but not the Wondfo Pf/Pan RDT, and the *P*. *ovale* LDH gene was relatively well conserved among the subspecies. Therefore, malaria diagnosis might be difficult using only RDTs, especially for *P*. *ovale* infections. The present results in the study could provide more aspects for producing better RDTs with significantly improved sensitivity for *P*. *ovale*.
